# The Association of EEG μ Rhythm Phase and Power with TMS-Assessed Cortical Excitability States

**DOI:** 10.3390/s25237187

**Published:** 2025-11-25

**Authors:** Wenshu Mai, Xinyu Zhao, Panli Chen, Yuezhuo Zhao, He Wang, Xin Wang, Zhipeng Liu, Jingna Jin, Tao Yin

**Affiliations:** 1 Institute of Biomedical Engineering, Chinese Academy of Medical Sciences and Peking Union Medical College, Tianjin 300192, China; wenshumai@126.com (W.M.); zhaoxy@pumc.edu.cn (X.Z.); panli_chen2022@163.com (P.C.); yuezhuozhao200209@163.com (Y.Z.); whe19882006@126.com (H.W.); wangxin121926@163.com (X.W.); lzpeng67@163.com (Z.L.); 2 The State Key Laboratory of Advanced Medical Materials and Devices, Tianjin 300192, China; 3 Tianjin Key Laboratory of Neuromodulation and Neurorepair, Tianjin 300192, China

**Keywords:** closed-loop TMS, electroencephalography, μ rhythm, phase, power, cortical excitability state

## Abstract

The efficacy of transcranial magnetic stimulation (TMS) is influenced by the brain’s real-time activity state. This study aimed to investigate the correlation between cortical excitability states and EEG features, specifically the phase and power of the sensorimotor μ rhythm. We developed a high-precision real-time phase prediction algorithm based on a Long Short-Term Memory (LSTM) network and constructed a closed-loop TMS system dependent on EEG phase and power. Thirty healthy subjects were recruited for single-pulse TMS experiments. Motor evoked potentials (MEPs) and TMS-evoked potentials (TEPs) were recorded simultaneously to assess cortical excitability states triggered in real time based on different EEG phase and power features. The results demonstrated no significant correlation between the μ rhythm phase and the amplitudes of MEPs or most TEP components. In contrast, pre-stimulus μ rhythm power showed a significant positive correlation with MEP amplitude. Under high-power conditions, the amplitude of the late P180 component in the sensorimotor cortex was significantly enhanced. The early-to-mid components (N15-N100) of the global mean field potential (GMFP) also exhibited significantly increased amplitudes. This study found that, compared to phase, EEG μ rhythm power exhibits a more significant correlation with TMS-assessed cortical excitability states. This finding provides a key basis for developing EEG power-dependent closed-loop TMS methods to enhance the efficacy of TMS modulation.

## 1. Introduction

Transcranial magnetic stimulation (TMS), a non-invasive physical stimulation technique that modulates brain neural activity via pulsed magnetic fields, faces challenges of unstable individual responses and the need for improved efficacy in broad clinical applications. Studies have reported that the modulatory effects of TMS may be related to the instantaneous state of brain neural activity at the time of stimulation. Electroencephalography (EEG) signals, with their millisecond-level temporal resolution, can capture the instantaneous dynamics of brain activity. Leveraging this advantage of EEG, researchers have begun to explore its combination with TMS to develop TMS methods triggered by brain states [1,2,3]. The sensorimotor μ-rhythm, an 8–14 Hz oscillation originating from the sensorimotor cortex during eyes-open rest, has been closely linked to cortical excitability regulation. Some studies, by triggering TMS at specific phases of the sensorimotor μ rhythm, have observed specific enhancement of corticospinal excitability [4]. These advances mark an important shift in TMS technology from fixed stimulation paradigms towards dynamic, individualized stimulation paradigms.

TMS methods triggered by brain states are built upon neurophysiological research. Studies indicate that the phase and power of the sensorimotor μ rhythm are two key indicators reflecting dynamic changes in cortical excitability. Animal experiments show that neuronal firing rates are highly correlated with the phase of local field potentials, with different oscillation phases corresponding to systematic differences in the excitability of neuronal populations. Simultaneously, in vivo experiments confirm that the level of μ rhythm power is directly related to the degree of synchronization of inhibitory interneuron population activity, which can modulate the response threshold of cortical neurons to external inputs [2]. These findings provide an important physiological basis for understanding the relationship between EEG features and cortical excitability states.

Based on this, existing studies typically assess cortical excitability states through motor evoked potential (MEP) amplitude and analyze its correlation with the phase and power of the EEG μ rhythm. However, the results are inconsistent. Some findings show that TMS applied at the trough of the EEG wave evokes significantly larger MEP amplitudes compared to the peak [5], while other results indicate no significant effect of EEG phase on MEP amplitude [6]. Furthermore, some TMS studies have observed that higher pre-stimulus μ rhythm power is associated with higher MEP amplitudes, a finding that contradicts expectations based on physiological rationale. Clearly, consistent conclusions have not yet been reached regarding the correlation between EEG μ rhythm phase/power and TMS-assessed cortical excitability states.

Based on the current research status outlined above, this study utilized Long Short-Term Memory (LSTM) networks [7,8], which can capture long-term dependencies and complex nonlinear features in time series, to develop a μ rhythm phase estimation algorithm based on LSTM EEG signal prediction. Using this algorithm, a high-accuracy closed-loop TMS system was constructed. Healthy subjects were recruited for closed-loop single-pulse TMS experiments. Cortical excitability states were then reflected using two indicators: MEPs and TMS-evoked potentials (TEPs), which can measure cortical excitability [9,10,11,12]. The correlations between cortical excitability states and EEG phase and power at the time of TMS were analyzed. This study aims to investigate the relationship between the two EEG features (phase and power) and cortical excitability states, provide a basis for EEG feature selection in closed-loop TMS methods, promote the establishment of this method and its clinical application, and advance the development of individualized neuromodulation techniques.

## 2. Methods

### 2.1. Subjects

This experiment recruited 36 healthy subjects aged between 20 and 29 years (average age 22.7 ± 2.4 years, 18 males). The inclusion criterion required that the resting motor threshold (RMT) measured while wearing the EEG cap did not exceed 83% of the maximum stimulator output (MSO). This criterion was established because the experimental procedure involved administering TMS during EEG recording and required acquisition of motor evoked potentials (MEPs) at 120% of RMT. Six subjects were excluded due to failure to meet the RMT criterion, resulting in a final cohort of 30 participants. All subjects in this experiment had not taken any psychotropic medications and had no prior history of central nervous system disorders or head injuries. The study was conducted in accordance with the safety guidelines for TMS [13,14,15,16]. All participants tolerated the experimental procedures well, and no adverse events were reported. Prior to the experiment, participants were required to obtain sufficient sleep in order to maintain an optimal mental state throughout the procedure. All participants were fully informed of the study’s purpose and procedures and provided written informed consent. This study was approved by the Ethics Committee of the Institute of Biomedical Engineering, Chinese Academy of Medical Sciences and Peking Union Medical College.

### 2.2. Experimental Set-Up

**Figure 1:**
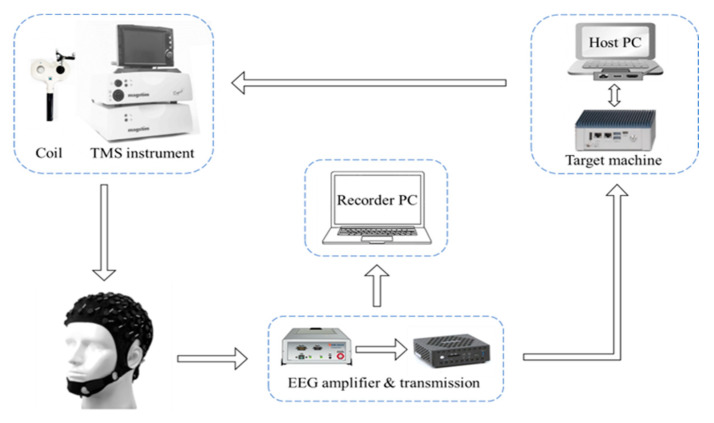
Schematic diagram of a closed-loop TMS system dependent on EEG phase. The μ rhythm phase of the EEG in the sensorimotor area was calculated in real time. When the μ rhythm phase of the EEG at the current moment met the preset conditions, the TMS pulse output was triggered.

The closed-loop TMS system operates based on the EEG phase (Figure 1) and consists of five main components: the EEG amplifier, the TMS device, the host computer, the target machine, and the recorder computer, with coil positioning monitored by a neuronavigation system (Brainsight, Rogue Inc., Edmonton, AB, Canada) to ensure targeting accuracy. An EEG-compatible TMS stimulator (Magstim Rapid^2^, Magstim Ltd., Whitland, UK) equipped with a figure-of-eight coil was employed. A 64-channel EEG was recorded using a TMS-compatible sintered Ag/AgCl ring electrode cap (EasyCap GmbH, Herrsching, Germany) and an EEG amplifier (Brain Products GmbH, Munich, Germany). The amplified EEG signals were routed through a TurboLink module (Brain Products GmbH, Gilching, Germany) to the target machine (a dedicated real-time computer, Speedgoat GmbH, Bern, Switzerland) and the Recorder PC using the UDP protocol. The target machine was connected to the host computer via Ethernet and was controlled by a Simulink Real-Time model (MATLAB, Natick, MA, USA, R2022a) for real-time extraction of the EEG phase and comparison with predefined conditions. Upon meeting the preset criteria, a TTL-level signal was generated from the target machine’s output port to trigger the delivery of the TMS pulse. The Recorder PC was responsible for the acquisition and storage of EEG data.

### 2.3. EEG Signal Processing

**Figure 2:**
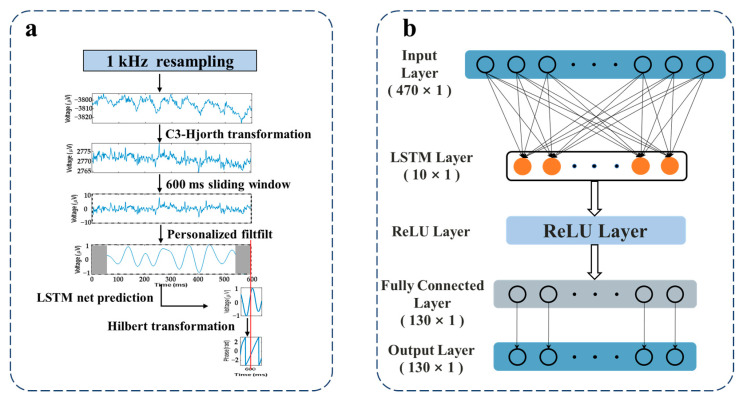
EEG μ rhythm phase extraction algorithm. (**a**) Phase prediction flowchart, including using the Hjorth transform to enhance μ rhythm signal-to-noise ratio, detrending to remove DC offsets, applying an individualized frequency domain filter for zero-phase spatial filtering and distortion edge correction, utilizing an LSTM network for forward signal prediction, and employing the Hilbert transform to compute the phase. (The red line indicates the TMS trigger timing). (**b**) The LSTM model architecture comprises five layers: an input layer (470 × 1), an LSTM layer (10 × 1), a ReLU layer, a fully connected layer (130 × 1), and an output layer (130 × 1). (The black dots indicate the omitted LSTM units).

The real-time phase extraction method for the EEG μ rhythm is illustrated in Figure 2a. Initially, the EEG signals acquired at 5 kHz were resampled at 1 kHz to align with the 1 ms fixed fundamental sample time step processing rate of the real-time model in the closed-loop TMS system. To enhance the signal-to-noise ratio (SNR) of μ rhythm, the EEG signal from the C3 channel was spatially filtered using the Hjorth transformation with a spatial filter composed of FC1, FC5, CP1, and CP electrodes. The transformation is defined by the following equation:

(1) $$L_{C3}=V_{C3}-\frac{1}{4}\sum {(V}_{FC1}+V_{FC5}+V_{CP1}+V_{CP5})$$

where $V_{C3}$ represents the potential at the central electrode C3 and $V_{FC1}$, $V_{FC5}$, $V_{CP1}$, and $V_{CP5}$ denote the potentials at the respective surrounding electrodes. $L_{C3}$ is the spatially filtered potential at the C3 channel. A 600 ms sliding window was used to define the analysis epoch, which was updated in real time with a step size of 1 ms upon acquiring new data. Within this window, the signal was first detrended to remove DC offsets. Then, the signal was filtered using a 128th-order zero-phase frequency-domain filter designed using the Hamming window method, centered at the individual μ rhythm frequency band ($f_{ind}$ ± 2 Hz), where $f_{ind}$ was determined from the power spectrum of the individual’s resting-state EEG. The frequency response of the filter is provided in Supplementary Material S1. To mitigate the edge distortions caused by frequency-domain filtering, a 65 ms long signal at both ends of the filtered signal was discarded, retaining an effective signal length for subsequent prediction. An individualized LSTM network was then employed to predict the time-domain signal within a 470 ms sliding window, generating a 130 ms prediction segment. The phase value of this predicted segment was obtained via Hilbert transform, and the phase value at the midpoint of the segment was extracted for further analysis. In this experimental set-up, to minimize inter-stimulus interference, the interval between consecutive stimuli was maintained at no less than 3 s. The individualized LSTM model was trained separately using 5 min resting-state EEG data from each participant and consisted of an input layer, an LSTM layer, a ReLU layer, a fully connected layer, and an output layer (Figure 2b). The LSTM model was optimized using the adaptive moment estimation (Adam) optimizer. The loss function was defined as the Mean Squared Error (MSE), calculated using the following formula:

(2) $$MSE=\frac{1}{N}\sum_{i=1}^{N}{\left(y_{i}-{ŷ}_{i}\right)}^{2}$$

where $y_{i}$ and ${ŷ}_{i}$ represent the true and predicted values, respectively. The maximum number of training epochs was set to 400.

### 2.4. EEG and Electromyography (EMG) Recording

EEG and EMG acquisitions were performed in the electrophysiology laboratory and the room was kept quiet during recording. EEG data were recorded with a 64-channel BrainAmp EEG system (Brain products GmbH, Munich, Germany). The impedance of all the electrodes was kept below 5 kΩ in the experiment. In this study, resting-state EEG data and EEG-synchronized TMS signals were collected at sampling rates of 1 kHz and 5 kHz, respectively. Surface electromyography of the first dorsal interosseous muscle was recorded with Ag/AgCl electrodes (Myoquick Matrix Line-Micromed Srl, Mogliano Veneto, Italy), with the ground electrode placed on the pisiform and the EMG signal sampling rate being 32768 Hz.

### 2.5. Experimental Session

**Figure 3:**
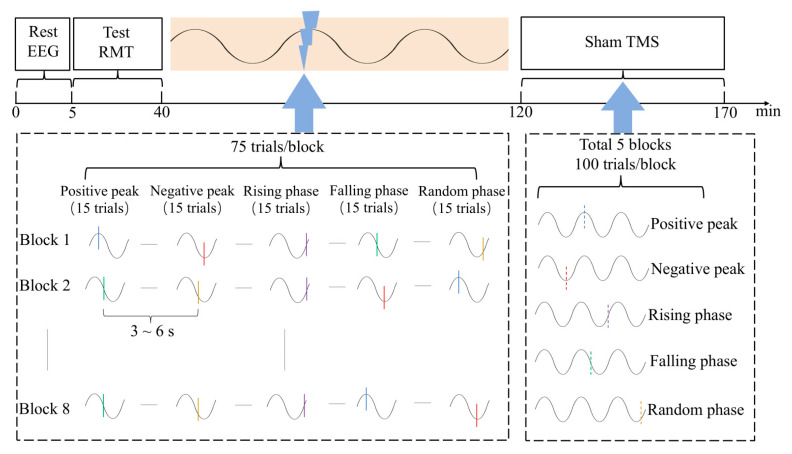
Experimental sessions. Initially, 5 min of eyes-open resting-state EEG signals were recorded to identify the peak frequency of the individualized μ rhythm and to train a personalized LSTM model for forward signal prediction. Subsequently, a closed-loop TMS experiment targeting specific phases of μ rhythm was conducted, during which EEG and EMG signals were simultaneously recorded under five different conditions: μ rhythm positive peak, negative peak, rising phase, falling phase, and random phase. Finally, to validate the accuracy of real-time μ rhythm phase triggering, five additional EEG recording conditions—comprising both triggered and unstimulated trials—were implemented. (The colored lines (blue, red, purple, green, yellow) indicate the five TMS trigger conditions: positive peak, negative peak, rising phase, falling phase, and random phase. The corresponding dashed lines of the same colors denote the sham stimulation trials for each of the five phase conditions. The two blue arrows indicate the detailed implementation session for the real TMS and sham stimulation block).

The experiment consisted of three parts. During EEG recording, the subjects were asked to fixate on the “+” sign in front of their eyes. The experimental procedure is shown in Figure 3. In the first part of the experiment, a 5 min eyes-open resting-state EEG signal was recorded. In the second part, eight blocks of single-pulse TMS were administered, each consisting of 75 pulses. These included 15 pulses targeting each of the five phase conditions: positive peak, negative peak, rising phase, falling phase, and random phase. The order of the phase conditions was randomized across blocks. The phase features of the signal were extracted according to the Hilbert transform. The stimulation intensity of TMS was 120% RMT, the stimulation target was the left motor cortex hotspot, and the interval between two adjacent pulses was set to no less than 3 s. After the experiment, the interpulse interval of the positive peak trigger group was 3.7 ± 1.1 s, the interpulse interval of the negative peak group was 3.5 ± 0.8 s, the interpulse interval of the rising phase trigger group was 3.5 ± 0.7 s, and the interpulse interval of the falling phase trigger group was 3.6 ± 1.0 s. The interval between two adjacent pulses in the random phase group was fixed at 3s. In the third part, five blocks of sham TMS were administered. During these blocks, only the EEG trigger timings were marked without delivering actual TMS pulses. Each block consisted of 100 triggers. Blocks 1 to 4 involved phase-dependent sham TMS targeting the μ rhythm, specifically triggered at the positive peak, negative peak, rising phase, and falling phase of the μ rhythm, respectively. Block 5 served as a phase-independent sham TMS control.

### 2.6. Pre-Stimulus Power

For each TMS trial, a 1000 ms pre-stimulus EEG epoch was extracted and downsampled to 1 kHz. To enhance the signal-to-noise ratio (SNR) of μ rhythm, the C3 channel signal was spatially filtered using the Hjorth transformation with FC1, FC5, CP1, and CP electrodes. Linear detrending was then applied to the resulting signal to remove DC offsets. Power spectral density (PSD) was estimated via Burg’s autoregressive model (model order = 100, frequency resolution = 1 Hz) applied to each 1000 ms epoch. The μ-band (8–14 Hz) power was computed as the mean PSD within the individualized frequency range ($f_{ind}$± 2 Hz), where $f_{ind}$ was determined from resting-state spectra.

### 2.7. MEP Analysis

The MEP amplitude evoked by each TMS stimulus was extracted for analysis. Specifically, EMG data segments spanning from −100 to 100 ms relative to the TMS onset were isolated. The MEP amplitude was defined as the peak-to-peak amplitude of the EMG signal within a time window of 20 to 40 ms after the TMS pulse. In this study, the EMG signal recorded during the interval from −100 to −5 ms relative to TMS was used as the baseline. The root mean square (RMS) value of the baseline signal was calculated across all trials, and 2.5 times the standard deviation (SD) of this RMS value was established as the threshold for determining muscle contraction or relaxation. Trials in which the baseline muscle activity exceeded the threshold were excluded from further analysis to ensure the purity of the TMS-evoked MEPs [17,18]. On average, 1% of trials were excluded per participant.

### 2.8. TEPs of Left Sensorimotor Cortex

TMS-EEG signals were analyzed using the TESA toolkit [19]. TMS-EEG data were first segmented, retaining the time period from 1000 ms pre-TMS to 1000 ms post-TMS. Then, the signal from 2 ms before to 10 ms after TMS pulse was interpolated three times, and the residual TMS noise and eye movement interference were removed by independent component analysis. Then, the 1–100 Hz bandpass filtering and 49–51 Hz bandpass filtering were performed. The components of TEPs (N15 (10–20 ms), P30 (20–40 ms), N45 (35–55 ms), P60 (45–75 ms), N100 (70–120 ms), and P180 (130–200 ms)) were extracted from the region of interest (ROI) composed of five leads (C3, FC5, FC1, CP5, CP1). In addition, to analyze the global differences in brain activity, the whole brain mean field potential (GMFP) was calculated.

(3) $$GMFP\left(t\right)=\sqrt{\left[\frac{\sum_{i}^{k}{\left(V_{i}\left(t\right)-V_{mean}\left(t\right)\right)}^{2}}{k}\right]}$$

where k is the number of channels, $V_{i}$ is the voltage measured by channel i, and $V_{mean}$ is the average potential of all included channels [20,21].

### 2.9. Statistics

Data are summarized using descriptive statistics and reported as mean ± SD. All statistical analyses were conducted using SPSS (version 21.0), Prism 8, and MATLAB (version 2022a). The amplitudes of each MEP across the positive peak, negative peak, rising phase, falling phase, and random phase groups were overlaid, averaged, and subsequently normalized using the Min–Max Normalization method. Normality of the data was evaluated using the D’Agostino–Pearson test. Since the data did not follow a normal distribution, statistical comparisons were performed using the Friedman test, followed by Dunn’s post hoc test for pairwise comparisons [22,23].

Subsequently, MEP amplitudes were categorized into three groups based on the magnitude of pre-stimulation power, and statistical analysis was carried out using the same non-parametric approach. Furthermore, stimulation power levels were divided into three groups according to MEP amplitude, and the power values across these groups were also analyzed using the aforementioned method.

To further investigate the relationship between stimulation power and MEP amplitude, both variables were treated as continuous measures. The Shapiro–Wilk test confirmed that both variables were normally distributed; therefore, Pearson correlation analysis was employed to assess the association between power and MEP amplitude for each individual.

Finally, the amplitudes of TEP components within the ROI were grouped according to phase and stimulation power, and the same statistical procedures were applied. In all analyses, the significance level was set at *p* < 0.05 unless otherwise specified.

## 3. Results

### 3.1. Online Phase-Triggered EEG-TMS

**Figure 4:**
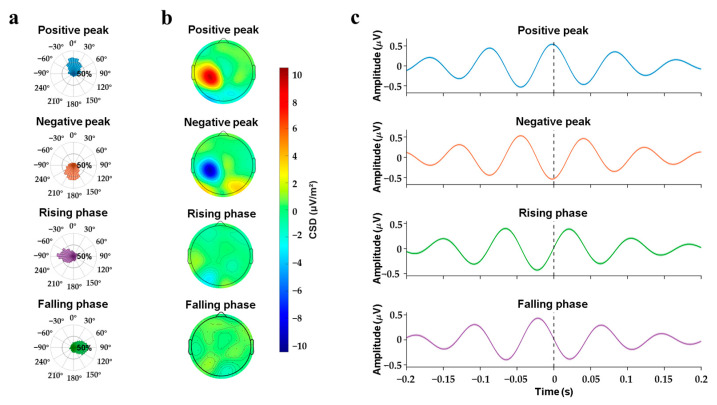
Phase-locking precision under sham TMS conditions was evaluated for four target phases: 0°, 180°, −90°, and 90°. (**a**) Actual phase angle at the target moment. (**b**) Mean CSD plots of the triggered non-stimulated four conditions, amplitudes indicated by the color bar. (**c**) Superimposed average of trials of the four trigger conditions, the black dashed line shows the timing of TMS trigger, and the shadow indicates ± 1 standard error of the mean (SEM). (The shadow is hardly visible due to the very small SEM, hence the band representing ±1 SEM ap-pears extremely narrow).

On average, the phase-dependent closed-loop TMS technique effectively delivered stimulation at the preset μ rhythm phase of the EEG (Figure 4). Real-time triggered non-stimulus trials were analyzed to confirm that all four targeted phases were accurately reached across subjects. Specifically, the positive peak was measured at 9.47° ± 60.25°, the negative peak at 170.18° ± 60.24°, the rising phase at −86.81° ± 64.54°, and the falling phase at 88.91° ± 63.79°.

The non-uniformity of the phase distribution across all four conditions was statistically confirmed using the V-test (*p* < 0.001 for all cases), indicating a significant concentration around the expected phases. Further analysis of the current source density (CSD) transformed EEG signals revealed that the neural activity corresponding to the positive peak, negative peak, rising phase, and falling phase of μ rhythm was localized to the sensorimotor cortex of the left hemisphere, as shown in Figure 4b. Additionally, the phase estimation method employed in this study was validated using the AR-based algorithm previously reported in the literature [24], with detailed results provided in Supplementary Material S2. These results demonstrate that the phase extraction algorithm developed in this experiment accurately identifies the phase of μ rhythm in the sensorimotor region and achieves higher accuracy compared to the AR-based phase estimation method.

### 3.2. Phase-Modulation

**Figure 5:**
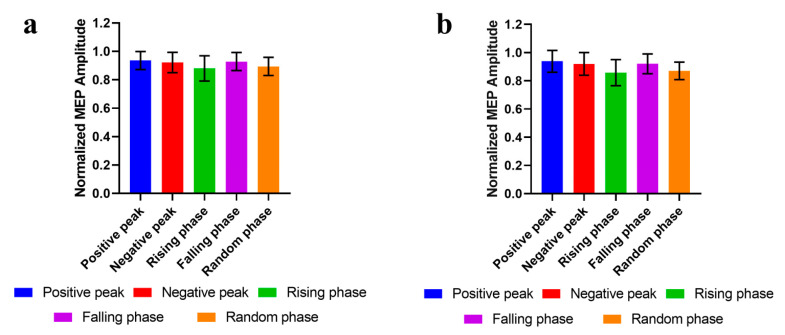
The μ rhythm phase effects on MEP amplitudes. (**a**) MEP amplitudes of all subjects (mean ± SD). (**b**) MEP amplitudes in subjects with μ rhythm SNR greater than 5 dB in the sensorimotor area (mean ± SD).

The normalized MEP amplitudes of the five phase conditions were as follows: 0.94 ± 0.07 at the positive peak, 0.93 ± 0.08 at the negative peak, 0.88 ± 0.09 at the rising phase, 0.93 ± 0.07 at the falling phase, and 0.89 ± 0.06 at the random phase (Figure 5a). The MEP amplitudes of each group were treated as the dependent variable and subjected to Friedman statistical analysis. The results showed no significant differences among the groups (χ^2^(4) = 7.33, *p* = 0.119), indicating that the phase of the EEG μ rhythm had no modulatory effect on the MEP amplitudes. Subsequently, considering that the accuracy of EEG phase estimation is positively correlated with the signal-to-noise ratio of μ rhythm phase, we calculated the signal-to-noise ratio for each subject and selected subjects with a signal-to-noise ratio higher than 5 dB to calculate the MEP amplitudes of each phase condition. Following the Friedman statistical analysis, no significant differences in MEP amplitudes were observed among the groups (χ^2^(4) = 6.86, *p* = 0.144) (Figure 5b).

**Figure 6:**
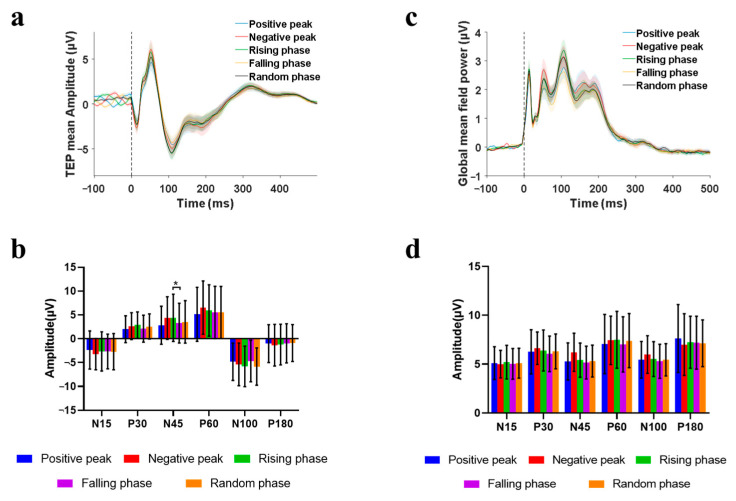
Phase-dependent TEPs in sensorimotor cortex. (**a**) Grand-averaged TEP waveforms (mean ± SEM) within the ROI across five phase groups, and shaded bands indicate ± 1 SEM. (**b**) Mean amplitudes of N15, P30, N45, P60, N100, and P180 components of the ROI across five phase groups (mean ± SD), * *p* < 0.05. (**c**) GMFP waveforms (mean ± 1 SEM) across five phase groups, and shaded bands indicate ± 1 SEM. (**d**) Mean amplitudes of N15, P30, N45, P60, N100, and P180 components of GMFP across five phase groups (mean ± SD), * *p* < 0.05.

Grand-averaged TEP waveforms (mean ± SEM) from −100 to 500 ms peri-stimulus were plotted for all the five phase-locking conditions within the ROI (Figure 6).

Grand-averaged TEP waveforms (mean ± SEM) within the ROI were analyzed across the five phase conditions, spanning from −100 ms (pre-stimulus baseline) to 500 ms post-stimulation (Figure 6a). Peak amplitudes of six characteristic TEP components were quantified for each phase condition. The Friedman test revealed a significant main effect of phase on the N45 amplitude (χ^2^(4) = 12.99, *p* = 0.0113). Post hoc analysis with Dunn’s test demonstrated that the amplitude at the negative peak was significantly higher than that at the falling phase (*p* = 0.0329). (Figure 6b).

GMFP waveforms (mean ± SEM) from −100 ms (pre-stimulus baseline) to 500 ms post-stimulation are shown in Figure 6c. The amplitudes of the components of GMFP after different phases of stimulation are extracted. Following the Friedman statistical analysis, no significant differences in amplitudes of the components of GMFP were observed among the groups (Figure 6d).

### 3.3. Power-Modulation

**Figure 7:**
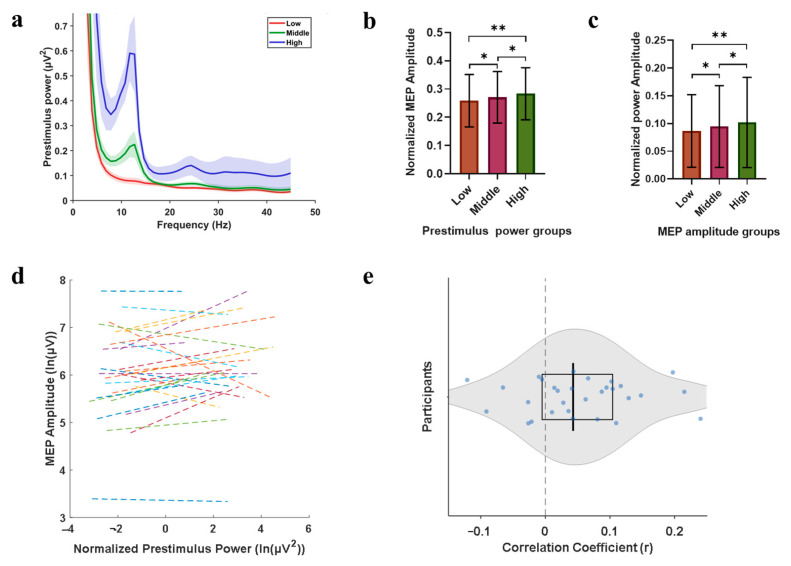
Modulation of MEP amplitudes by sensorimotor μ-rhythm power. (**a**) Group-averaged μ-band PSD across power groups (high/medium/low), expressed as mean ± SEM. (**b**) Normalized MEP amplitudes stratified by power groups, expressed as mean ± SD, * *p* < 0.05, ** *p* < 0.01. (**c**) Normalized μ-rhythm PSD values categorized by MEP amplitude groups (high/medium/low), expressed as mean ± SD, * *p* < 0.05, ** *p* < 0.01. (**d**) Linear correlation between the log amplitude of PSD and the log amplitude of MEP across all individuals(Each colored dashed line indicates an individual participant.). (**e**) Distribution of individual correlation coefficients. Each point represents one participant (*n* = 30). (The shaded area indicates the data distribution; the box and internal line indicate the interquartile range and median).

Based on the power magnitude of each subject during TMS, the power values were classified into three groups: high power, middle power, and low power (Figure 7a), confirming distinct EEG power levels. Normalized MEP amplitudes were calculated for each power group. Normality was confirmed via D’Agostino–Pearson tests (low power group: K^2^ = 3.689, *p* = 0.158; medium power group: K^2^ = 3.262, *p* = 0.196; high power group: K^2^ = 4.261, *p* = 0.119). As all *p*-values exceeded the significance level of 0.05, the data for all three groups were considered normally distributed. A repeated-measures one-way ANOVA with Geisser–Greenhouse correction revealed significant between-group differences (F (1.289, 37.39) = 9.204, *p* = 0.0023) (Figure 7b). The results of pairwise comparison showed that the amplitude of MEPs of the middle power group (*p* = 0.024) and the high power group (*p* = 0.008) was significantly higher than that of the low power group, and the amplitude of MEPs in the high power group was significantly higher than the middle power group (*p* = 0.036).

Then, we divided each subject into three groups according to the amplitude of TMS-evoked MEPs each time, namely low MEPs group, middle MEPs group and high MEPs group, and calculated the corresponding μ rhythm normalized power mean value of each group (Figure 7c). There were significant differences in power among the three groups (F (1.329, 38.55) = 8.711, *p* = 0.003). The results of pairwise comparison showed that compared with the low MEPs group, the PSD was significantly higher of the middle MEPs group (*p* = 0.045) and the high MEPs group (*p* = 0.009), and the power was significantly higher of the high MEPs group than of the middle MEPs group (*p* = 0.031).

Furthermore, the log values of power and MEP were used as continuous variables. The Shapiro–Wilk test showed that the log variable of power and the log variable of MEP both fit the normal distribution. Pearson correlation analysis was used to calculate the correlation between the two variables (Figure 7d,e). At the overall level, μ rhythm power has no linear correlation with MEPs (*p* = 10^−24^, r = 0.0761). The relevant conditions of each subject are shown in Supplementary Material S3.

**Figure 8:**
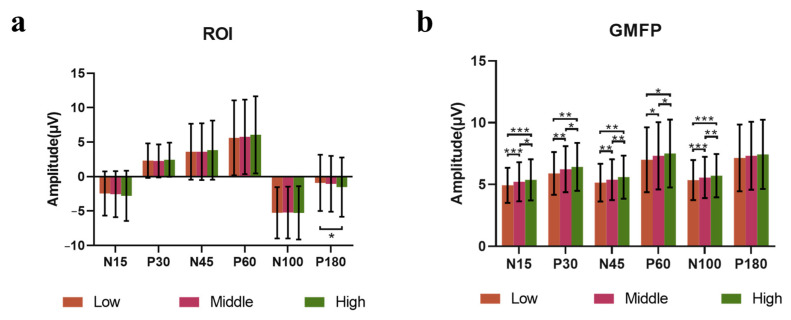
Results of TEPs across high, middle, and low power groups. (**a**) Mean amplitudes of N15, P30, N45, P60, N100 and P180 components of ROI, * *p* < 0.05; (**b**) Mean amplitudes of N15, P30, N45, P60, N100, and P180 components of GMFP, * *p* < 0.05, ** *p* < 0.01, *** *p* < 0.001.

Based on the ROI, the amplitudes of components after stimulation with high, middle and low power were extracted. Friedman statistical analysis showed that the amplitudes of TEP had a significant difference at N180 component (χ^2^(2) = 7.724, *p* = 0.0210). With Dunn’s test, it was shown that the amplitude in the high power condition was significantly higher than that in the low power condition (*p* = 0.0295). (Figure 8a).

There were significant differences in the GMFP amplitudes among the high, middle and low power groups of N15, P30, N45, P60, and N100. The results of pairwise comparison showed that the GMFP amplitude of the middle power group and the high power group was significantly higher than that of the low power group, and the high power group was significantly higher than the middle power group (Figure 8b), and the significant parameters are shown in Supplementary Material S4.

### 3.4. Consistency of Coil Position During Closed-Loop TMS

During the whole TMS experiment, we recorded the distance deviation and Angle deviation between the center of the coil and the hotspot during each pulse stimulation. Under the five conditions, the distance deviation is within the range of (0.972 ± 0.454) mm, and the Angle deviation is within the range of (2.897 ± 1.093)°. RM one-way ANOVA showed that there was no significant difference in distance deviation (F (3.421, 99.21) = 1.483, *p* = 0.219) and Angle deviation (F (2.722, 78.94) = 1.833, *p* = 0.153) among the groups (see Table 1). It is shown that our experimental results are not caused by a shift in the position of the coil during stimulation during the experiment.

## 4. Discussion

To clarify the performance of EEG phase and power as features for closed-loop TMS, this study first leveraged the advantage of LSTM in dynamic time series signal prediction to establish a more accurate EEG phase estimation method and a closed-loop TMS system dependent on EEG features. Using this system for experiments, the results from MEPs and TEPs revealed no significant and consistent relationship between the EEG μ rhythm phase at the time of TMS and the excitability state of the motor cortex. Only the effect of TMS on the excitability state of the motor cortex at 35–55 ms when applied synchronously with the negative peak was significantly stronger than when applied during the falling phase. Furthermore, while Pearson correlation analysis revealed no strong linear relationship, the analysis based on power groupings revealed a significant effect: higher power states were associated with increased cortical excitability. This result was consistent across MEPs and early-to-middle TEP components (10–120 ms) across the whole brain. This suggests that the association between μ rhythm power and excitability can be reliably detected through a group-level analysis, presenting a contrast to the less consistent effects observed for the EEG phase.

Previous research by Zrenner’s team [25,26] found that TMS applied at the trough of the μ rhythm evoked significantly higher MEPs compared to the peak, which contradicts our findings. Differences in how our experiment was conducted may explain these results. All studies by Zrenner’s team screened subjects based on resting-state μ rhythm power or signal-to-noise ratio, excluding subjects with low μ rhythm power or SNR. We found that for subjects recruited without limiting μ rhythm power or SNR indices, the modulatory effect of the μ rhythm phase was not significant. Notably, similar to Zrenner’s team, we further selected a subject population with high μ rhythm SNR and still did not find a modulatory effect of the μ rhythm phase on MEP and TEP amplitudes. Furthermore, a subgroup analysis across SNR levels showed no phase modulation for MEPs, the TEP components of the ROI, or the GMFP amplitude (Supplementary Material S5). This evidence suggests that the findings regarding phase modulation are not caused by SNR. Another difference from Zrenner’s team’s experiment is the stimulus intensity. Zrenner’s team selected a TMS intensity corresponding to an MEP amplitude of 1 mV, while our experiment used 120% RMT. Judging from the amplitude of the MEPs we collected (<1 mV), we used a slightly lower stimulus intensity. Literature reports that the relationship between the μ rhythm and MEPs may be influenced by stimulus intensity, which could be one reason for the discrepancy in our results. Alternatively, some studies are consistent with our results: the μ rhythm phase does not modulate motor cortex excitability. Studies using intracranial recordings of potential changes in humans have also reported no μ phase dependent modulation of neuronal firing in the human somatosensory cortex [27]. This indicates that although the regulatory effect of the μ rhythm on external stimulation can be observed in animals, this phenomenon may not exist in humans. It may be due to the volume conduction effect of the head, leading to the diffusion of EEG signals recorded on the scalp and low signal-to-noise ratio, making this effect undetectable.

Compared to the results for μ rhythm phase, the literature on the effect of μ rhythm power on cortical excitability is relatively consistent. It generally reports that high power states are associated with increased excitability, which is also consistent with our study results. However, higher μ rhythm power is classically associated with increased neural inhibition, which aligns with the pulsed inhibition theory of the μ rhythm. This contradicts our results and those reported in the literature. The reason for this may be that almost all studies in this field focus on the transient PSD of the C3 electrode located in the sensorimotor area. Studies on the correspondence between EEG electrodes and brain functional areas show that the C3 electrode in most individuals is located in the somatosensory and motor areas of the postcentral gyrus, rather than the primary motor area of the precentral gyrus. This makes it possible that the C3 montage we used is more sensitive to radial sources from the top of the postcentral gyrus (S1) than tangential sources from the precentral gyrus (M1). Therefore, the EEG signals we analyzed may originate from a different population of neurons (S1) than the one probed by MEPs (M1). Given that tight sensory–motor connectivity involves substantial feedforward inhibition, it is possible that increased power leads to inhibition in the somatosensory cortex (S1) but rhythmically releases M1 from general sensory–motor inhibition. Future studies should explicitly investigate the role of the S1-M1 interaction on the transient PSD to more clearly prove our speculation. However, this hypothesis requires validation through future studies employing direct measurements of functional connectivity between S1 and M1 regions, for example, by calculating coherence between EEG electrodes overlying S1 and M1 [10]. Nevertheless, this study, in conjunction with other reports, supports the existence of a relationship between μ rhythm power and cortical excitability. Specifically, high power groups exhibit increased cortical excitability, a result that appears to be relatively stable and reproducible.

Our experiments tried to eliminate the influence of factors other than EEG power and EEG phase. First, all our experiments were conducted at the same time of day (1 p.m. to 5 p.m.) to prevent the influence of circadian effects [28] on the results. Second, all experiments were performed by the same operator, who was familiar with the TMS instrument and navigation and positioning system and had extensive practical experience to ensure the consistency of the experimental procedure between and within subjects. Finally, our experiments were all conducted under the guidance of precise positioning navigation. After excluding trials with large coil position errors during stimulation, the error associated with each TMS output coil placement was minimal, ensuring that the experimental results were not influenced by inaccuracies in stimulus placement. These measures taken in our experiment ensure, as far as possible, that the experimental results are due to the factors we are concerned with.

Our study used individual EEG features to control the timing of magnetic pulse output, representing a closed-loop TMS technique for stimulation timing [29,30]. The results reliably confirmed that high power identifies a state of increased TMS-assessed cortical excitability states, suggesting that developing EEG power-dependent closed-loop TMS timing stimulation technology is expected to enhance its effect. However, in this study, we only established the more commonly studied EEG phase and built a TMS technology dependent on EEG phase. We did not use an EEG power-dependent TMS system to validate the results online, which becomes an important research topic to be carried out in our next step. Another limitation of our study is that we only conducted TMS experiments at a 120% RMT stimulation intensity [13,31]. Some studies have reported [24] that the relationship between EEG phase and TMS-assessed cortical excitability states is dependent on stimulus intensity, where MEP amplitude can be modulated by the EEG μ rhythm phase only at weaker stimulation intensities, but not at higher intensities. Therefore, we cannot rule out whether there is still no correlation between them at other stimulation intensities, which requires more experiments to analyze and verify. Finally, this study used suprathreshold stimulation intensity during TMS, allowing MEPs to be evoked in peripheral muscles for assessing the degree of cortical excitability. However, the related changes in re-afferent input generated by evoked muscle twitch may affect the modulation of TMS cortical responses [32,33]. Given that TEPs can still be recorded during subthreshold stimulation [34], future studies could use TEPs to investigate the relationship between the two during subthreshold TMS.:

## Figures and Tables

**Table 1:** Distance and angle deviations of the center of the stimulating coil from the target point of five phase conditions.

Phase (°)	Target Error (mm) (mean ± std)	F Statistic	*p* Value	Angular Error (°) (mean ± std)	F Statistic	*p* Value
0	0.955 ± 0.570	1.483	0.219	2.729 ± 1.801	1.833	0.153
180	0.965 ± 0.645			2.637 ± 1.842		
−90	0.953 ± 0.604			2.787 ± 1.879		
90	0.982 ± 0.601			2.700 ± 1.799		
random	1.001 ± 0.625			2.701 ± 1.891		

## Data Availability

The original contributions presented in the study are included in the article, further inquiries can be directed to the corresponding authors.

## Supplementary Materials

The following supporting information can be downloaded at: https://www.mdpi.com/article/10.3390/s25237187/s1.
