# Supplementary material for: The Association of EEG μ Rhythm Phase and Power with TMS-Assessed Cortical Excitability States
Source: Sensors (Basel). 2025 Nov 25;25(23):7187. doi: 10.3390/s25237187 (PMC12694099; doi:10.3390/s25237187)
Supplement: Supplementary file 1 [file sensors-25-07187-s001.zip › sensors-3952825-supplementary.pdf]

## Supplementary Material S1

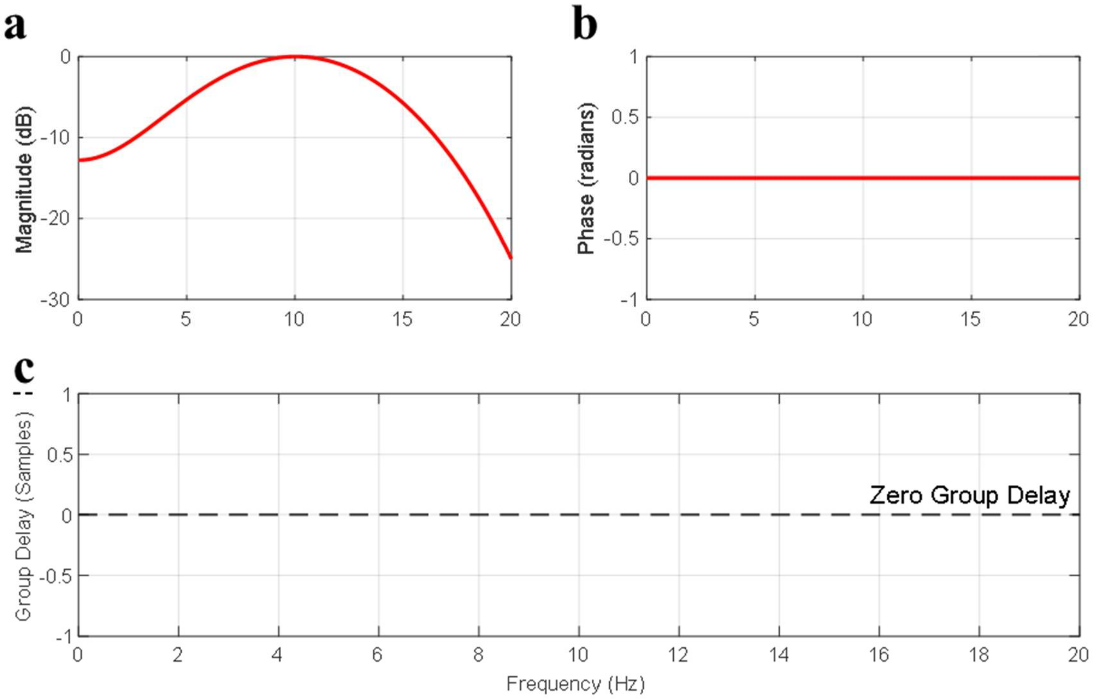


Figure S1. Frequency response of the 128th-order zero-phase FIR filter.
(a) Magnitude response. (b) Phase response. (c) Group delay.

## Supplementary Material S2

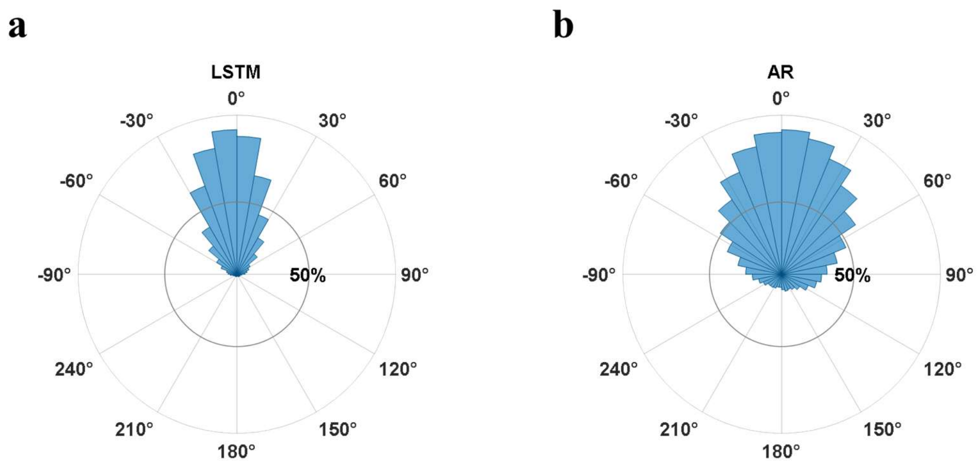


Figure S2. Phase estimation errors from offline analysis of resting-state EEG data. (a) Error between the estimated phase and the ground-truth phase using the LSTM-based phase estimation method. (b) Error between the estimated phase and the ground-truth phase using the AR model-based phase estimation method.

Means and standard deviations of the error between the estimated phase and the ground-truth phase

| Method | Mean（°） | SD（°） |
|---|---|---|
| LSTM | 3.0 | 42.6 |
| AR | 4.3 | 67.8 |

**Supplementary Material S3**

The relevant conditions between PSD and MEPs of each subject

| Subjects | Pearson Correlation Coefficient | P value |
|---|---|---|
| S1 | 0.2539 | $2.809 \times 10^{-10}$ |
| S2 | -0.2448 | $1.358 \times 10^{-9}$ |
| S3 | 0.2304 | $1.179 \times 10^{-8}$ |
| S4 | 0.2059 | $3.635 \times 10^{-7}$ |
| S5 | 0.1835 | $6.046 \times 10^{-6}$ |
| S6 | 0.1754 | $1.623 \times 10^{-5}$ |
| S7 | 0.1682 | $3.470 \times 10^{-5}$ |
| S8 | -0.1622 | $6.579 \times 10^{-5}$ |
| S9 | -0.1614 | $7.180 \times 10^{-5}$ |
| S10 | 0.1601 | $8.155 \times 10^{-5}$ |
| S11 | 0.158 | $1.012 \times 10^{-4}$ |
| S12 | 0.1536 | $1.581 \times 10^{-4}$ |
| S13 | 0.1319 | $1.000 \times 10^{-3}$ |
| S14 | 0.1256 | $2.000 \times 10^{-3}$ |
| S15 | 0.1238 | $2.000 \times 10^{-3}$ |
| S16 | 0.1166 | $4.000 \times 10^{-3}$ |
| S17 | 0.0882 | $3.100 \times 10^{-2}$ |
| S18 | 0.0867 | $3.400 \times 10^{-2}$ |
| S19 | 0.0864 | $3.500 \times 10^{-2}$ |
| S20 | 0.0809 | $4.800 \times 10^{-2}$ |
| S21 | -0.0719 | $7.900 \times 10^{-2}$ |
| S22 | -0.0716 | $8.000 \times 10^{-2}$ |
| S23 | -0.0674 | $9.900 \times 10^{-2}$ |
| S24 | 0.058 | $1.482 \times 10^{-1}$ |
| S25 | -0.0487 | $2.341 \times 10^{-1}$ |
| S26 | 0.0429 | $2.944 \times 10^{-1}$ |
| S27 | 0.0335 | $4.127 \times 10^{-1}$ |
| S28 | -0.0127 | $7.565 \times 10^{-1}$ |
| S29 | -0.0028 | $9.458 \times 10^{-1}$ |
| S30 | -0.0005 | $9.911 \times 10^{-1}$ |

## Supplementary Material S4

The statistical analysis results of GMFP for the N15, P30, N45, P60, and N100 groups

| Peak | Comparison | T value | P value |
|---|---|---|---|
| N15 | Low vs Middle | 5.120 | <0.0001 |
| | Low vs High | 4.824 | <0.0001 |
| | Middle vs High | 2.766 | 0.0112 |
| P30 | Low vs Middle | 3.745 | 0.0013 |
| | Low vs High | 3.675 | 0.0014 |
| | Middle vs High | 2.246 | 0.0305 |
| N45 | Low vs Middle | 3.067 | 0.0052 |
| | Low vs High | 3.932 | 0.0006 |
| | Middle vs High | 3.623 | 0.0013 |
| P60 | Low vs Middle | 2.662 | 0.0167 |
| | Low vs High | 3.213 | 0.0047 |
| | Middle vs High | 2.352 | 0.0323 |
| N100 | Low vs Middle | 3.844 | 0.0005 |
| | Low vs High | 4.340 | 0.0001 |
| | Middle vs High | 3.004 | 0.0063 |

## Supplementary Material S5

As shown in Figure S3a, there was no significant difference in MEP amplitude among the phase conditions in the high, medium and low SNR groups. As shown in Figure S3b, each TEP component of ROI did not show significant differences among the phase conditions in the high, medium and low SNR groups. As shown in Figure S3c, there was no significant difference in GMFP amplitude among the phase conditions in the high, medium and low SNR groups.

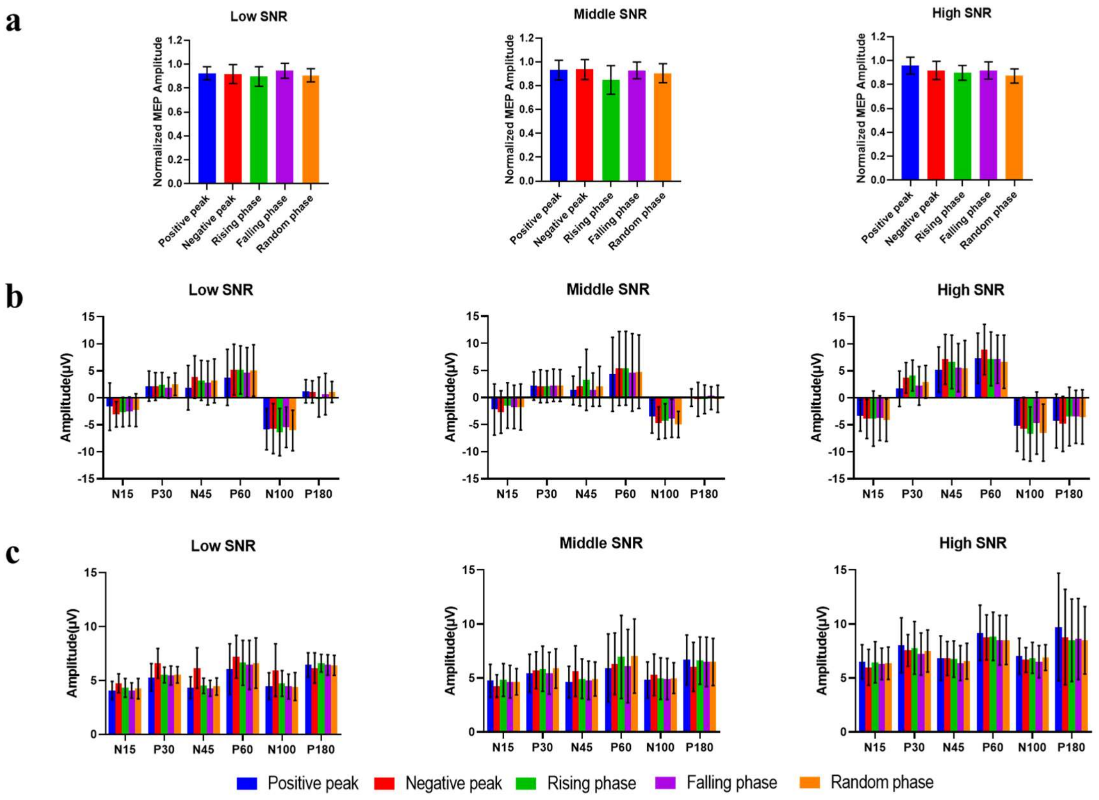


Figure S3. Phase-dependent measurements in the high, medium and low SNR groups.
(a) MEP amplitudes among five phase conditions in the high, medium and low SNR groups.
(b) Mean amplitudes of N15, P30, N45, P60, N100, and P180 components of the ROI among five phase conditions in the high, medium and low SNR groups. (c) Mean amplitudes of N15, P30, N45, P60, N100, and P180 components of GMFP among five phase conditions in the high, medium and low SNR groups (mean ± SD), $**p < 0.01$.
